# Editorial: COVID and psychotropics 2024: lessons learnt and future directions for research

**DOI:** 10.3389/fpsyt.2026.1800334

**Published:** 2026-03-03

**Authors:** Patricia Di Ciano, Soumitra Das, Paz Toren, Norbert Scherbaum

**Affiliations:** 1 Institute for Mental Health Policy Research, Centre for Addiction and Mental Health, Toronto, ON, Canada; 2 Department of Pharmacology and Toxicology, University of Toronto, Toronto, ON, Canada; 3 Dalla Lana School of Public Health, University of Toronto, Toronto, ON, Canada; 4 Campbell Family Mental Health Research Institute, Toronto, ON, Canada; 5 Department of Psychiatry, University of Melbourne, Melbourne, VIC, Australia; 6 Ramat-Chen Brüll Mental Health Center, Tel-Aviv District, Clalit Health Services Community Division, Tel Aviv, Israel; 7 Gray Faculty of Medical&Health Sciences, Tel Aviv University, Tel Aviv, Israel; 8 LVR-University Hospital Essen, Department of Psychiatry and Psychotherapy, Medical Faculty, University of Duisburg-Essen, Essen, Germany

**Keywords:** alcohol, mental health, pandemic, stress, Treatment

COVID-19 is a contagious virus caused by the coronavirus SARS-CoV-2. It spread rapidly throughout the world and the World Health Organization declared COVID-19 a global pandemic on March 11, 2020. After that, the world saw unprecedented measures to contain the spread of the disease, including quarantine and isolation, measures that persisted for years. During the pandemic, enormous efforts went into developing treatments for the virus, and efforts to market a vaccine eventually yielded treatments with some success.

Efforts began to understand the virus and implications of quarantines and lockdown. At the start of the pandemic it was postulated that alcohol use may increase due to the stress of the lockdowns, but conversely that restrictions on access may reduce alcohol intake (1). Results of some syntheses reveal heterogeneity in results, with alcohol consumption decreasing in some countries while the proportion of problematic drinking and heavy drinking episodes may have increased (2). The present Research Topic sheds some light into this question. Tran et al. found that, in Lithuania, the heaviest drinkers drank more during COVID-19. In contrast, contrary to prediction, the lowest drinkers did not decrease their alcohol intake. In another study, Roser et al. found a slight increase in use of gabapentinoids and opioid analgesics (>5%) during the COVID-19 pandemic and a decrease in cannabis use (>5%). Future studies should unravel the nuances of heterogeneity in populations, regions and different types of drug use.

Lockdowns and isolation can be understandably stressful (3). Problems with many syntheses of the effects of COVID-19 on stress is the fact that these studies are retrospective in nature. In the present Research Topic, Bae et al. used a novel approach of using a wearable device to track stress of patients. They found that perceived stress scores were higher in patients with COVID-19. Thus, COVID-19 can be stressful as a pandemic but it can also lead to stress in people with the illness. Increased psychological stress and anxiety can also have secondary effects such as declines in performance. Indeed, Zegarra-Valdivia et al. found that global cogntion, as assessed with the Addenbrooke’s Cogitive Examination (ACE) was lower in people with a history of COVID-19. Further, Shao et al. found that disease severity, quality of life and mental health were worse in psoriasis patients during COVID-19, highlighting the need for mental health supports by dermatologists in the treatment of psoriasis. Future studies will to track the progress of these patients and determine best ways to intervene.

The scope of the COVID-19 pandemic and severity of the illness lead to a number of efforts to find treatments for the illness. A search of cinicaltrials.gov at the time of writing this Editorial revealed over 10,000 hits, highlighting the significant effort that has gone into the management of this illness. A search of COVID-19 and ‘lithium’ yielded two studies that investigated the effects of lithium on long COVID. In the present Research Topic, Avni et al. found that lithium was protective for COVID-19, which is novel. Further, there has been some suggestion that some treatments for illnesses may worsen COVID-19 symptoms, and Albarahi et al. found that COVID-19 was not worse in people receiving treatment with clozapine. Future studies should continue to find novel ways to treat and manage COVID-19.

In sum, the present Research Topic offers insights into the effects of COVID-19 on a variety of measures of wellnesses and provides new evidence for an impact of COVID-19 on the use of other substances. A number of questions remain, about the nuances of the effects of COVID-19, and also the effect of COVID-19 on comorbid illnesses. The COVID-19 pandemic may be behind us, but the lessons of quarantine and isolation continue, and will likely continue to do so for years to come.
